# The Link between Parental Support and Adolescent Negative Mood in Daily Life: between-Person Heterogeneity in within-Person Processes

**DOI:** 10.1007/s10964-020-01323-w

**Published:** 2020-09-30

**Authors:** Loes H. C. Janssen, Bernet M. Elzinga, Bart Verkuil, Manon H. J. Hillegers, Loes Keijsers

**Affiliations:** 1 Department of Clinical Psychology, Leiden University, Leiden, the Netherlands; 2 Leiden Institute for Brain and Cognition (LIBC), Leiden, the Netherlands; 3 Department of Child and Adolescent Psychiatry/Psychology, Erasmus University Medical Centre–Sophia Children’s Hospital, Rotterdam, the Netherlands; 4 Department Developmental Psychology, TSB, Tilburg University, Tilburg, the Netherlands

**Keywords:** Experience sampling method (ESM), Daily life, Parental support, Adolescent negative mood, Heterogeneity, Within-family level

## Abstract

Lack of parental support is related to more adolescent negative mood. However, little is known about how fluctuations of parental support relate to fluctuations of negative mood within adolescents in daily life. The current study aimed to elucidate these processes at a day to day micro-level and examined to which extent adolescents would differ in the association between perceived parental support and adolescent negative mood. The sample consisted of 242 Dutch adolescents (*M*_age_ = 13.82, 63.2% female) who completed ecological momentary assessments of 3 weeks 3 months apart. Results from the multilevel regression analyses showed that, on average, adolescents experienced higher levels of negative mood on days when they perceived their parents to be less supportive. Substantial individual differences were found in this association, however, these were partially explained by the level of depressive symptoms and perceived parental intrusiveness. These findings suggest that advice on parental support should be tailored to the unique characteristics of the adolescent.

## Introduction

Adolescence is an important developmental period with several challenges and changes. Even though most adolescents cope successfully with the biological, psychological and social changes, it does make adolescence not only a window of opportunity, but also a vulnerable period for the onset of internalizing problems (Dahl et al. 2018). Empirical studies and reviews have suggested that a lack of parental support may be a proximal cause of internalizing problems (e.g., Pinquart 2017), but how this process unfolds in daily life remains unclear. Mood swings and negative daily mood have recently been identified as a precursor for the development of internalizing problems (Maciejewski et al. 2014), and these day to day fluctuations in negative mood may be linked to parent-child interactions in daily life (e.g., Keijsers et al. 2016). The vast majority of research on parenting and adolescent well-being is based on analysis of data at the aggregate level, resulting from longitudinal designs with macro timescales (i.e., years) and classical retrospective self-report measures, while the underlying mechanisms of adolescent development and parenting processes more specifically, are dynamic, person-specific, and take place in the daily flow of life (Keijsers and Van Roekel 2018). The results of existing studies with longer time intervals may therefore not provide us with information about how daily fluctuations of support and negative mood influence each other on a smaller time scale (Keijsers and Van Roekel 2018). Investigating these underlying social processes at a more micro-level (i.e., hours, days) within persons may yield relevant insights into the building blocks of longer term mental health development (Boele et al. 2019). Therefore, by using ecological momentary assessments (EMA; Stone and Shiffman 1994) the current study aimed to examine the more proximal associations between experienced parental support and adolescent daily negative mood within a person in the daily flow of life and assess individual differences. Furthermore, four factors were examined to explain possible heterogeneity (i.e., adolescent gender, severity of adolescent depressive symptoms, perceived intrusiveness of parents, and overall social support).

### Parental Support and Adolescent Negative Mood

Parents are one of the more proximal factors affecting adolescent development (Sameroff 2000). Even though friendships gain in importance during adolescence, parental support remains to be one of the key sources of emotional well-being for adolescents (e.g., Furman and Buhrmester 1992). Many studies have been conducted on parental support and internalizing problems (e.g., Pinquart 2017) and focused on relative differences between families. Recently, a systematic review of 46 studies found only two studies which investigated the micro processes between perceived parental support and adolescent negative mood at the within-person level (Boele et al. 2019). However, results of statistical analyses at the group level do not necessarily contain information on how processes operate at the level of the individual (e.g., Hamaker et al. 2015), and this is also true for parenting studies (Keijsers 2016). In fact, because group level and individual level tap into different sources of variance, the two analytical levels answer different research questions: Whereas between-person associations at the group level shed a light on how individuals differ and who is at risk, the within-person associations at the individual level highlight when a given individual is at risk (Keijsers and Van Roekel 2018). The two studies that assessed the within-person association between perceived parental support and negative mood used the same dataset of 8 weeks of daily diary data from 47 adolescents (aged 8 to 13 years old). The first study detected that more negative mood was associated with less parental support of mothers (Bai et al. 2016). The second study found that early adolescents reported more negative mood on days that they perceived their parents (both fathers and mothers) to be less supportive (Reynolds et al. 2016). It remains unclear whether this also applies to older adolescents. Furthermore, the two studies used daily diary data of both negative mood and parental support. Early adolescents had to indicate how they felt during a day at the end of each day. However, mood can fluctuate throughout the day and recall bias might have affected these negative mood scores. The current study therefore adds to the few within-person studies by including older adolescents and using a more intensive longitudinal data collection method EMA (Stone and Shiffman 1994) 8 times a day during 3 separate weeks 3 months apart, to reduce adolescents’ recall bias in reporting negative mood. The first aim was therefore to examine the micro processes of parenting and study whether and how fluctuations of parental support would be related to fluctuations of negative mood within adolescents in daily life.

### Individual Differences in the Association between Parental Support and Adolescent Negative Mood

Theoretically, it is increasingly acknowledged that children and adolescents may respond in different ways to parenting (e.g., Keijsers et al. 2016; Sameroff 2010). The association between parental support and adolescent negative mood thus may vary from adolescent to adolescent. Even though such heterogeneity in the processes has been acknowledged in several parenting theories (e.g., Pluess and Belsky 2010), as well as the broader category of ecological theories (Bronfenbrenner and Morris 2006; Sameroff 2010), not many studies have investigated such hypotheses regarding differential effects (or heterogeneity) in the within-person processes (Boele et al. 2019). Assessing this heterogeneity in proximal processes requires more intensive longitudinal data, such as daily diaries or experience sampling, which have been relatively scarce (Van Roekel et al. 2019). By combining EMA with daily diary data and multilevel analyses, this study addressed whether adolescents differ in the association between daily parental support and daily negative mood. Moreover, to obtain a more in-depth understanding of why some adolescents respond positively to parental support, whereas other respond in terms of negative mood the current study tested four plausible moderating factors (gender, severity of adolescent depressive symptoms, parental intrusiveness, and general levels of social support).

A first factor that might explain these individual differences is gender. In general, girls are more likely to experience negative affect than boys (Zahn-Waxler 2000) and it was tested whether lack of parental support affected negative mood more in girls than boys. Second, the association between parental support and negative mood in daily life may be different for adolescents with more depressive symptoms than for adolescents with less depressive symptoms. Negative behaviors (e.g., social withdrawal, excessive reassurance seeking) shown by adolescents with substantial depressive symptoms might result in parents withdrawing support, also known as support erosion (Slavin and Rainer 1990), which may impact adolescent negative mood. Thirdly, adolescents can perceive parental support differently based on the intrusiveness (e.g., snooping or asking inappropriate questions; Hawk et al. 2008) of parents. When parents are perceived as intrusive, support may relate to negative, rather than positive outcomes for the adolescent (e.g., Dietvorst et al. 2017). Finally, the presence of social support of others (e.g., friends) might be another relevant factor (Furman and Buhrmester 1992) that could buffer against a lack of parental support. Whether or not adolescents can rely on a different source(s) of support than their parents might also affect how sensitive they are to parental support in daily life.

## The Current Study

The current study aimed to elucidate the within-person association between perceived parental support and adolescent negative mood in daily life and examined individual differences in these within-person associations. Based on previous studies (Bai et al. 2016; Reynolds et al. 2016) and reviews (Boele et al. 2019), it was hypothesized that for the average adolescent, lack of parental support at a given day would be associated with more adolescent negative mood on that day. Secondly, substantial heterogeneity was expected to be found in these associations explained by moderators. It was expected that a lack of parental support would be more strongly related to adolescent negative mood in girls than boys; that the association between parental support and negative mood would be stronger for adolescents who show more depressive symptoms than adolescents who show less depressive symptoms; that the association between parental support and adolescent negative mood would be less strong, or even reversed, for adolescents who report more parental intrusiveness than adolescents who report less parental intrusiveness; and that relying on another source of support might buffer the negative effect of a lack of parental support on adolescent negative mood, making the association less strong among adolescents with more social resources.

## Methods

### Sample and Procedure

Data were used from the “Grumpy or Depressed” project (Keijsers et al. 2015), a Dutch multi-method, longitudinal study using both questionnaires as EMA to differentiate normative grumpy behavior during puberty from the early signs of depression. In the study, 604 adolescents of 21 second to fourth classes (preparatory secondary school for college and university) of a high school in the south of the Netherlands participated (province Limburg). The project was composed of two phases; a screening phase and a longitudinal study on a subsample and was approved by the ethics committee of the Faculty of Social Sciences at the University Utrecht in 2014.

In September 2014, all parents were informed about the study during information evenings of the school and were asked to provide passive informed consent for the screening phase. The screening (labeled T0) took place during school hours on computers, and included adolescents completing an extensive online questionnaire of approximately 45 min.

Subsequently, parents and adolescents were contacted to participate in the longitudinal study composed of three measurement waves within one school year (labeled T1, T2, T3; 3-month intervals). Each wave entailed online questionnaires for parents and for adolescents, and an EMA measurement burst (Nesselroade 1991) among adolescents. The online questionnaire was sent four weeks before the start of the EMA and parents and adolescents were given seven weeks to complete this online questionnaire. Prior to the start of this longitudinal study, adolescents and their parents provided active informed consent, both for the assessments as for the use of the screening data.

Each EMA wave consisted of filling out questionnaires on their own smartphone using the mobile app MyPanel for seven consecutive days (21 days in total) between 8AM and 10PM. Written information on how to download and install the app was provided to adolescents on the last page of the online questionnaire. Before the start of each EMA week, researchers checked whether adolescents logged into the app and contacted adolescents via WhatsApp, phone or mail when this was not the case. Adolescents received eight questionnaires randomly per day (56 in total) signaled by a notification and were instructed to fill out the questionnaires as quickly as possible. All questionnaires included the same items on whereabouts, mood, and substance use. In the first questionnaire of each day two items were added on sleep, in the last questionnaire of each day nine items were added on feelings, delinquent behavior, and parenting throughout the day. The morning questionnaire expired after two hours and the evening questionnaire after four hours. The other six questionnaires throughout the day expired after 90 min. The questionnaires consisted of 23 items, including one open-ended question, and filling out the questionnaires took 1–2 min per questionnaire (average 2 min, *SD* = 6.2). The school gave permission for adolescents to fill out EMA questionnaires during school hours, yet, when it would interfere with their school tasks participants could silence their phone. Researchers monitored the EMA by checking daily whether adolescents completed questionnaires and sent messages regularly to the adolescents via WhatsApp on the project telephone to stimulate completing the questionnaires. Adolescents did not receive automatic reminders for the questionnaires, since this was not possible yet. As a token of appreciation, each adolescent received a gift voucher of €5,- for their participation and among these adolescents five iPad-mini’s (worth approximately €250) were raffled.

#### Inclusion

Inclusion criteria were owning a mobile phone and speaking and understanding Dutch. Of the 604 adolescents, 573 adolescents participated in the screening of which 44.1% boys and 55.9% girls. Of the screened adolescents (*n* = 573), 46.9% agreed to participate (*n* = 269) in the EMA study. Twenty-five adolescents were not able to participate because of organizational problems (i.e., phone did not work, withdrawal of consent). In total 244 adolescents filled out the first EMA wave, at the second EMA wave 186 adolescents participated (76.2%), and at the last wave 186 adolescents participated (76.2%). Only data of adolescents who completed any daily diary (questionnaire in the evening) containing the item on daily parental support were selected for this study. Two adolescents did not complete any evening questionnaire throughout the EMA and were therefore deleted from the data resulting in a final sample of 242 adolescents, of which 89 boys (36.8%) and 153 girls (63.2%) with a mean age of 13.82 (*SD* = 0.92). Of the 242 adolescents, 213 (88.0%) indicated living together with at least their biological mother and father, 8 (3.3%) indicated living with mother, 2 (0.8%) indicated living with father, 18 (7.4%) indicated a different living situation (i.e., parent and stepparent, alternating between father and mother), and the living situation of one adolescent was unknown (0.4%). Most adolescents 216 (89.3%) reported having at least one sibling. Furthermore, the majority of adolescents was born in the Netherlands (98.3%), two were born in other countries within Europe (0.8%), and one was born in Asia (0.4%). Reports of parents (*n* = 235 parents; 44 males, 191 females) on educational level were used as indicator of socioeconomic status in the Netherlands. Of the 235 parents, 11.9% reported lowest levels (lower vocational education), 41.3% intermediate (higher vocational education), and 44.3% high levels (college/university education).

#### Compliance

Since daily parental support was only assessed in the evening questionnaire, compliance rates were focused on this questionnaire. At the first week, 231 adolescents filled out 972 evening questionnaires (60.1% of the possible evening assessments) leading to an average of 4.21 (*SD* = 1.93) diaries out of 7 days per adolescent. If a daily parental support score was missing, daily negative mood of that day was not used. The daily negative mood scores of the first week were based on 5109 assessments, with an average of 22.12 (*SD* = 13.44) completed questionnaires per adolescent and 4.97 per day per adolescent. At the second week, 169 adolescents filled out 611 evening questionnaires (51.6% of the possible evening assessments) leading to an average of 3.62 (*SD* = 1.87) daily diaries out of 7 days per adolescent. The daily negative mood scores of the second week were based on 3394 assessments, with an average of 20.08 (*SD* = 14.76) completed questionnaires per adolescent and 5.00 per day per adolescent. At the third week, 156 adolescents filled out 618 evening questionnaires (56.6% of the possible evening assessments) leading to an average of 3.96 (*SD* = 2.06) daily diaries out of 7 days per adolescent. The negative mood scores of the third week were based on 3434 assessments, with an average of 22.01 (*SD* = 16.59) completed questionnaires per adolescent and 4.99 per day per adolescent. No participants were removed from the data based on compliance rates.

#### Missing data analysis

Little’s MCAR tests (1995) on the full data per wave (i.e., daily parental support, daily negative mood, depressive symptoms, perceived intrusiveness, and perceived social support) indicated that the pattern of missing data did not deviate from a MCAR pattern in each of the measurement waves (EMA T1, χ^2^ = 544.34, *df* = 540, *p* = 0.440; EMA T2, χ^2^ = 466.81, *df* = 484, *p* = 0.705; EMA T3, χ^2^ = 487.03, *df* = 490, *p* = 0.529; online questionnaires, χ^2^ = 54.31, *df* = 45, *p* = 0.161). A more in-depth analyses of the missing data revealed that some missing EMA assessments were due to technical issues (i.e., signaling beep was not loud enough and therefore sometimes missed). Moreover, the level of EMA compliance was unrelated to the adolescent’s depressive symptoms and level of perceived intrusiveness at T1, T2, and T3, and unrelated to social support at T0 (all *p*’s > 0.05). Little (1995) shows that multilevel models using ML estimation and including all available data results in unbiased estimates, already under conditions of MAR. Therefore, with a MCAR pattern of missing observations, and ML estimation, the proposed multilevel models should be able to result in unbiased estimates.

### Measures

#### Daily negative mood

Momentary negative mood during the three EMA weeks (T1, T2, T3) was assessed with three items which were rated eight times a day with answer categories ranging from 1 (*not)* to 7 (*very*). These items were selected from items used in earlier EMA studies to assess negative mood (e.g., Morris et al. 2010; Riediger et al. 2014). Daily negative mood was measured by the items: “I feel sad”, “I feel disappointed” and “I feel unhappy”. A mean score per day of these three items was calculated to create scale scores reflecting daily negative mood, with a higher score indicating more negative mood. A nested alpha for daily negative mood was calculated (Nezlek 2017). The complete dataset was used and resulted in nested α = 0.787 for EMA T1, nested α = 0.882 for EMA T2, and nested α = 0.883 for EMA T3. These nested alphas indicated good between-person reliability of this novel instrument for assessing daily negative mood. The omega coefficient, a coefficient of within-person reliability (Schuurman and Hamaker 2019), was additionally calculated per week by performing three multilevel confirmatory factor analyses in Mplus 8.3 (Muthén and Muthén 1998–2017). For EMA T1, the omega coefficient was 0.812, for EMA T2 0.807, and for EMA T3 0.864. These omega coefficients indicated good within-person reliability.

#### Daily parental support

Adolescents rated parental support once at the end of each day during the three EMA weeks (T1, T2, T3) by answering the question which was developed for this study: “My parents were warm or supportive today”. Answer categories ranged from 1 (*not)* to 7 (*very)*, and a higher score indicated more parental support that day. Confirmatory Factor Analyses (CFA) were performed in R (lavaan package) to assess the convergent validity of this novel daily parental support instrument against the subscale support of the well-established Network of Relationships Inventory (NRI; Furman and Buhrmester 1985). Appendix 1 in the Supplementary Materials provides model fit information. As expected, there was a significant positive correlation between the latent factors capturing parental support measured by the NRI and the latent factor capturing the average of the daily assessments (standardized estimates: T1 = 0.563; T2 = 0.490; T3 = 0.621). The intraclass correlation (ICC) of daily parental support was 0.504 suggesting that 50.4% of the variance in adolescent daily parental support was due to differences between adolescents, and the remainder 49.6% due to within-person fluctuations over time.

#### Depressive symptoms

In the online questionnaires (T1, T2, T3), adolescent depressive symptoms were assessed using the Dutch version of the Children’s Depression Inventory (CDI-I; Kovacs 1992; Timbremont et al. 2008). The CDI-I consists of 27 items consisting of three statements graded in order of increased severity from 0 to 2 that described how they were feeling the last two weeks (e.g., “I get sad from time to time/I get sad often/I’m always sad”). Answers were summed to obtain a total score and some items were reversed to ensure that a higher score indicated more depressive symptoms. The Dutch version of the CDI has shown good validity and reliability (Timbremont et al. 2008). Cronbach’s alphas in the three measurement waves for adolescent depressive symptoms ranged between 0.87–0.89. A person-mean score of the CDI-I scores on all three waves was calculated to represent adolescent depressive symptoms. Based on CDI-I cut-off scores (Kovacs 1992; Timbremont et al. 2008) at T1 90.1% of the sample reported little to none depressive symptoms (score 0–11), 4.5% subclinical (score 12–15) and 5.4% clinical (score > 16), at T2 89.1% reported little to none symptoms, 3.8% subclinical, and 7.1% clinical, and at T3 89.2% reported little to none symptoms, 4.8% subclinical, and 5.9% clinical respectively.

#### Perceived intrusiveness

Adolescent perceptions of parental intrusiveness were assessed in the online questionnaires (T1, T2, T3) with the Dutch translation of the intrusiveness subscale of the Level of Expressed Emotion scale (LEE: Hale et al. 2007). For the purpose of the study, the subscale was shortened to the following three items that had the highest factor loadings in the study of Hale et al. (2007): “Are always nosing into my business”, “Have to know everything about me” and “Are always interfering”. Answer categories ranged from 1 (*true*) to 4 (*not true)*, but were reverse coded before calculating a mean intrusiveness score per wave. A higher score indicated more perceived intrusiveness. A person-mean score on the intrusiveness subscale on all three waves was calculated to represent perceived intrusiveness. Between-person reliability, assessed with Cronbach’s alphas in the three measurement waves for perceived intrusiveness ranged between 0.86–0.92. Earlier studies in Dutch samples support the factorial validity of the full scale (e.g., Hale et al. 2007).

#### Perceived social support

In the screening questionnaire (T0), general social support perceived by adolescents was assessed using the subscale social support of the short version Utrecht Coping List (UCL; Schreurs et al. 1993). Adolescents indicated their reaction to bad things happening or having problems. The subscale consisted of six items (e.g., “Sharing their concerns with someone”) and answer categories ranged from 1 (*seldom or never*) to 4 (*very often*). A person-mean score of these six items was calculated to represent perceived social support and a higher score indicated more social support. Cronbach’s alpha for perceived social support was 0.86. Reliability and validity of the UCL in adolescents has been demonstrated in other studies (Schaufeli and Van Dierendonck 1992).

### Strategy of Analyses

Multilevel models (also known as linear mixed effects models; Hox et al. 2017) were specified in R (R Core Team 2019) Version 3.6.1, using the multilevel version 2.6 (Bliese 2016) package to test the hypotheses with ML estimation. Likelihood ratio tests were used to assess differences in fit of the models (following guidelines of Hox et al. 2017). For centering, guidelines proposed by Hoffman (2015) and Bolger and Laurenceau (2013) were followed. Level 1 predictors were person-mean centered and Level 2 predictors grand-mean centered.

A series of models were tested. First, an unconditional random intercept model was specified (Model 1) that splits the total variance in adolescent daily negative mood into stable between-person differences and within-person fluctuations. Second, to explain these within-person fluctuations in adolescent daily negative mood, a person-mean centered predictor (daily parental support) was added with fixed effects at the within-person level (Model 2) to the random intercept model (Model 1). This model captured the hypothesized within-person effects of daily parental support on daily negative mood for the average adolescent. Third, variation was allowed around the slope, to test the hypothesized heterogeneity between persons in the within-person effects of parental support on daily negative mood (Model 3). That is, instead of considering the within-person effect of daily parental support on daily negative mood to be the same across persons as in Model 2, it was modeled as a random effect that varies between persons in Model 3 and the association between the random intercept and random slope was also included in Model 3. To give insight into the effect sizes, the standardized effect (beta) per person was computed with the formula b*SD(X)/SD(Y) (Schuurman et al. 2016). Fourth, if such heterogeneity between persons was found based on improved model fit on a Likelihood ratio test, the level 1 random effects were predicted by adding grand-mean centered predictors as main effect as well as in interaction with daily parental support, namely a grand-mean centered score of gender (Model 4a), a grand-mean centered score of adolescent depressive symptoms on all three waves (Model 4b), a grand-mean centered score of perceived social support (Model 4c) and a grand-mean centered score of perceived intrusiveness on all three waves (Model 4d). The hypothesized moderating effect of each predictor was tested separately by adding a main effect of the predictor and interaction of the predictor with daily parental support both to Model 3. Fifth, all predictors (main effect of predictor and interaction between predictor and daily parental support) that significantly improved the model fit were then added together to the model and this model was the final model (Model 5).

Correlation structure corAR1 was added to take into account the time intervals of the study (Singer and Willett 2003). This structure was used since the days represent equally spaced time intervals. However, data from three waves with a three-month time interval was used and to correct for possible confounding influences thereof, the variable EMA week was added to the correlation structure in each model. Two-tailed tests with an α = 0.05 were used.

## Results

### Descriptive Statistics

Table 1 provides descriptive statistics and correlations. Initial differences between boys and girls were tested. Girls reported significantly more depressive symptoms than boys (*t* = −3.050, *df* = 231, *p* = 0.003; boys: *M* = 4.31, *SD* = 3.41; girls: *M* = 6.41, *SD* = 5.81), and more perceived social support (*t* = −4.867, *df* = 240, *p* < 0.001; boys: *M* = 2.15, *SD* = 0.50; girls: *M* = 2.54, *SD* = 0.65). No significant difference between boys and girls was found in perceived intrusiveness (*t* = 1.962, *df* = 231, *p* = 0.051; boys: *M* = 2.31, *SD* = 0.66; girls: *M* = 2.12, *SD* = 0.71). In daily life, no significant differences between boys and girls were found in daily negative mood (*t* = −1.426, *df* = 240, *p* = 0.155; boys: *M* = 1.29, *SD* = 0.52; girls: *M* = 1.41, *SD* = 0.68), and in daily parental support (*t* = −1.192, *df* = 240, *p* = 0.235; boys: *M* = 5.19, *SD* = 1.53; girls: *M* = 5.43, *SD* = 1.50).

**Table 1:** Descriptive statistics and bivariate correlations of study variables

Variables	Descriptives					Between-Person correlations					
	*n*	*M*	*SD*	Min	Max	1	2	3	4	5	6
Person level											
1. Gender	242	0.63	0.48	0.00	1.00						
2. Age	242	13.82	0.92	12.00	16.00	0.008					
3. Depressive symptoms	233	5.65	5.16	0.00	27.00	0.197**	0.121				
4. Parental intrusiveness	233	2.19	0.70	1.00	4.00	−0.128	0.058	0.280***			
5. Social support	242	2.40	0.63	1.00	4.00	0.300***	−0.020	−0.188**	−0.156*		
6. Person mean daily parental support	242	5.34	1.51	1.00	7.00	−0.021	−0.151*	−0.329***	−0.261***	0.205**	
7. Person mean daily negative mood	242	1.36	0.63	1.00	4.58	0.105	0.074	0.603***	0.236***	0.039	−0.298***

*p < 0.05; **p < 0.01; ***p < 0.001

### Baseline Model

Multilevel models were used to assess within-person fluctuations and heterogeneity of adolescent daily negative mood. In a first unconditional model (Model 1, Table 2 provides the results), the total variance in adolescent daily negative mood was partitioned into within-person over time fluctuations and stable between-person differences. The intraclass correlation of daily negative mood was 0.478, indicating that 47.8% of the variance in adolescent daily negative mood was due to differences between adolescents, and 52.2% due to within-person fluctuations over time.

**Table 2:** Results of Model 1, Model 2, and Model 3 on the relation between daily parental support and daily negative mood

	Model 1	Model 2	Model 3
Fixed effects: estimate *(SE)*			
Intercept	1.337*** (0.035)	1.337*** (0.035)	1.338*** (0.035)
Daily parental support		−0.031*** (0.009)	−0.031* (0.012)
Random effects			
Between person variance	0.231	0.230	0.231
Within person variance	0.252	0.250	0.244
Random effect variance			0.008
ICC	0.478	0.479	0.486
*N* individuals	242	242	242
*N* observations	2201	2201	2201

*p < 0.05; **p < 0.01***; *p* < 0.001

### Daily Parental Support and Daily Negative Mood

**Fig. 1:**
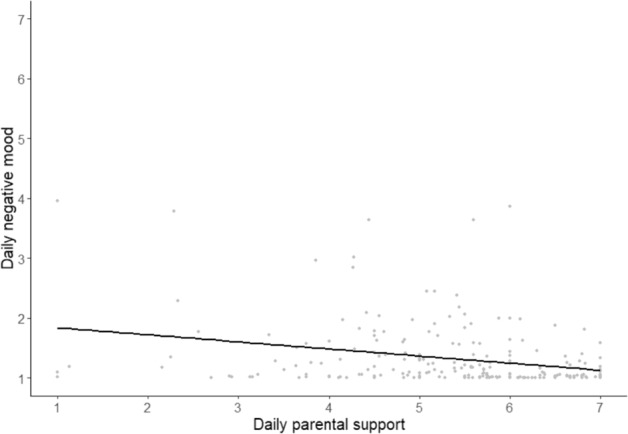
Between-person association between daily parental support and adolescent daily negative mood. Each dot represents one person, the line indicates overall association

In Model 2, the association between adolescent daily negative mood and daily parental support at the within-person level was tested. Adding the predictor improved the model fit compared to Model 1 (χ^2^(1) = 12.37, *p* < 0.001). Appendix 2 in the Supplementary Materials provides information on the model comparisons. In support of the hypothesis, results showed that, on average, adolescents report more negative mood on days when they perceived their own parents to be less supportive (*B* = −0.031, *SE* = 0.009, *df* = 1958, *t* = −3.521, *p* < 0.001) as shown in Fig. 1. Results are shown in Table 2.

### Heterogeneity between Adolescents

**Fig. 2:**
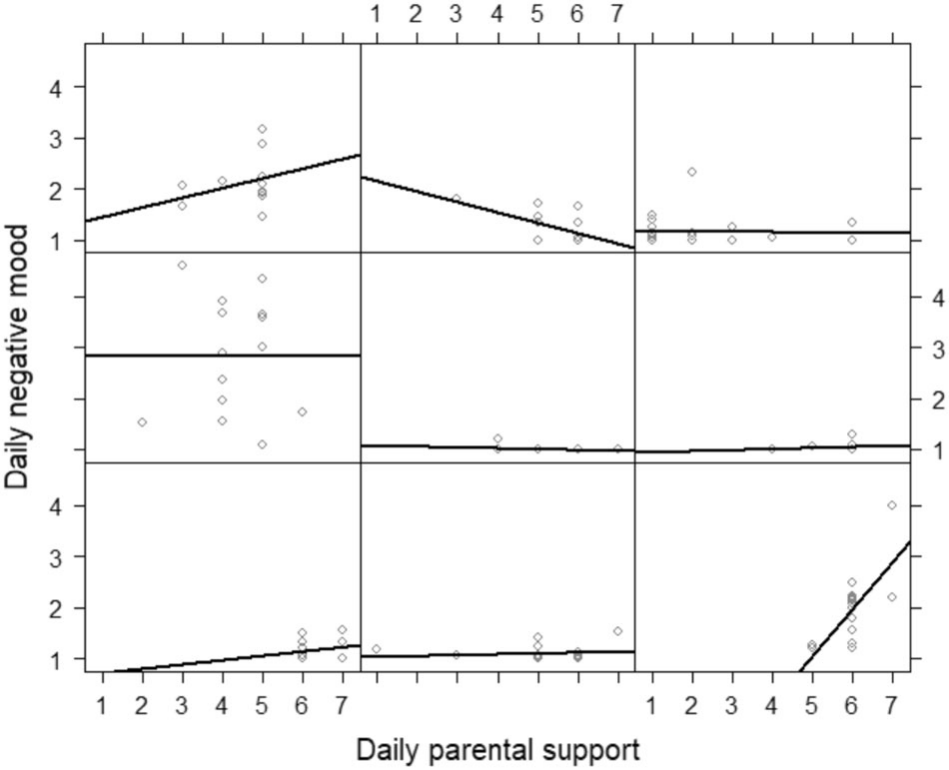
Within-person associations between daily parental support and adolescent daily negative mood of nine randomly chosen adolescents. Each panel represents one adolescent, each dot a measurement point

**Fig. 3:**
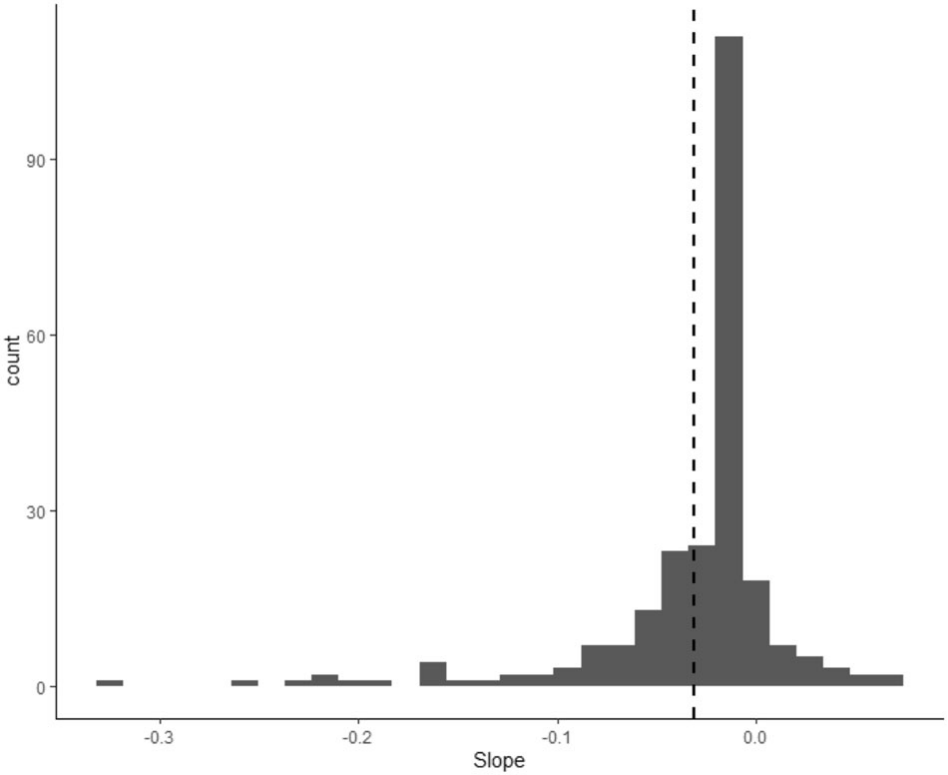
Range of unstandardized individual slopes of daily parental support related to daily negative mood in adolescents. Dashed line indicates the mean of the slopes

To assess heterogeneity between adolescents, a random slope was added allowing variation around the within-person effects of parental support on negative mood (Model 3), which improved the model fit compared to Model 2 (χ^2^(2) = 42.93, *p* < 0.001). Again, as indicated in Table 2, a significant within-person association between daily parental support and daily negative mood was found (*B* = −0.031, *SE* = 0.012, *df* = 1958, *t* = −2.585, *p* = 0.010). Moreover, in support of the hypothesis, across individuals variance (0.008) was found around the association between daily parental support and adolescent daily negative mood. Figure 2 displays these bivariate within-person associations for nine randomly chosen adolescents with a minimum of 10 observations per person. Figure 3 shows the distribution of all unstandardized individual slopes ranging between −0.320 and 0.073. The majority of adolescents (*n* = 218, 90.1%) reported more negative mood on days when they perceived their parents to be *less* supportive, while a minority of adolescents (*n* = 24, 9.9%) reported more negative mood on days when they perceived their parents to be *more* supportive. To provide a first insight into effect sizes, standardized effects (beta) per person were computed for adolescents with a minimum of 10 observations per person. These standardized effects ranged from −0.436 to 0.241. Following Cohen’s guidelines (1992) for effect size interpretation, the effect was moderately negative (−0.50 to −0.30) in 7.92% of the adolescents, small negative (−0.30 to −10) in 32.67%, small positive (0.10 to 0.30) in 8.91%, a weak effect was found (−0.10 to 0.10) in 36.63%, and for 13.86% standardized effects were missing due to no variance.

### Explaining Heterogeneity

To explain differences between adolescents with regard to the link between daily parental support and daily negative mood, gender (Model 4a), adolescent depressive symptoms (Model 4b), perceived social support (Model 4c), perceived parental intrusiveness (Model 4d) were added, and also an interaction term between these predictors and daily parental support. These models and model fit were compared to Model 3b. Some of the variables that were used to explain heterogeneity had missing values (96 observations of 20 adolescents) therefore these adolescents and observations were deleted from the data. Model 1 to Model 3 were repeated on the sample of *n* = 222 as Model 1b, Model 2b, and Model 3b to check whether deleting these observations influenced the results. This was not the case, results on the sample of *n* = 222 were comparable to the results on the sample of *n* = 242. Results of Model 1b to Model 3b can be found in Appendix 5 in the Supplementary Materials.

In contrary to the hypotheses, adding gender (Model 4a) and perceived social support (Model 4c) did not improve the model fit. Adding main and interaction terms of adolescent depressive symptoms (Model 4b) and perceived intrusiveness (Model 4d) did, which supported the hypotheses (model fit statistics are shown in Appendix 3 in the Supplementary Materials and model results are shown in Appendix 4 in the Supplementary Materials). Only main and interaction terms of adolescent depressive symptoms and perceived intrusiveness were therefore included in the final model (Model 5).

### Final Model

The model fit of Model 5 was significantly better compared to Model 3b (χ^2^(4) = 91.092, *p* < 0.001) and Table 3 provides results of Model 5. Fluctuations at the within-person level in daily parental support (*B* = −0.023, *SE* = 0.009, *df* = 1880, *t* = −2.490, *p* = 0.013) were still significantly linked to fluctuations in daily negative mood. The mean level of adolescent depressive symptoms (*B* = 0.063, *SE* = 0.006, *df* = 219, *t* = 10.926, *p* < 0.001) was also significantly linked to daily negative mood, but perceived parental intrusiveness was not related to daily negative mood after controlling for adolescent depressive symptoms. Thus, adolescents who reported more depressive symptoms also reported more negative mood in daily life, and adolescents reported more negative mood on days when they reported less parental support.

**Table 3:** Results of final Model 5 on the relation between daily parental support and daily negative mood and the moderating role of depressive symptoms, and perceived intrusiveness

	Model 5
Fixed effects: estimate *(SE)*	
Intercept	1.334*** (0.029)
Daily parental support	−0.023* (0.009)
Depressive symptoms	0.063*** (0.006)
Depressive symptoms*daily parental support	−0.008*** (0.002)
Perceived intrusiveness	0.038 (0.043)
Perceived intrusiveness* daily parental support	0.032* (0.013)
Random effects	
Between person variance	0.124
Within person variance	0.250
Random effect variance	<0.001
ICC	0.331
*N* individuals	222
*N* observations	2105

*p < 0.05; **p < 0.01; ***p < 0.001

The severity of adolescent depressive symptoms (*B* = −0.008, *SE* = 0.002, *df* = 1880, *t* = −4.527, *p* < 0.001) and perceived parental intrusiveness (*B* = 0.032, *SE* = 0.013, *df* = 1880, *t* = 2.434, *p* = 0.015) both moderated the within-person link between parental support and adolescent’s negative mood in daily life and thus explained parts of why this association differed between adolescents, explaining almost all random variation in Model 5.

**Fig. 4:**
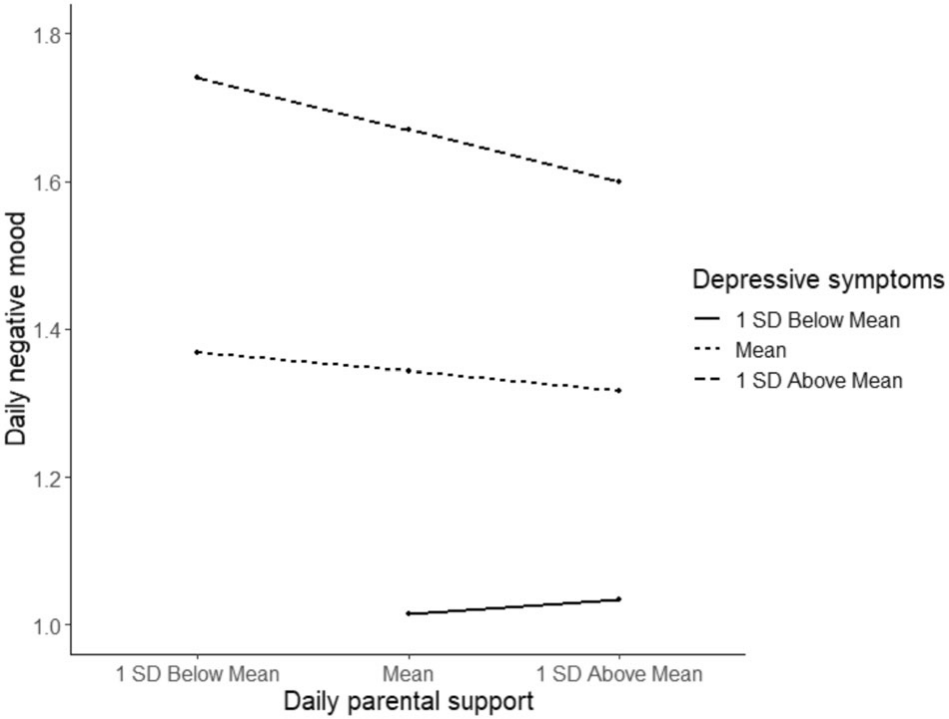
Simple slopes of daily parental support and daily negative mood for adolescents low or high in depressive symptoms based on (±1 Standard Deviation)

**Fig. 5:**
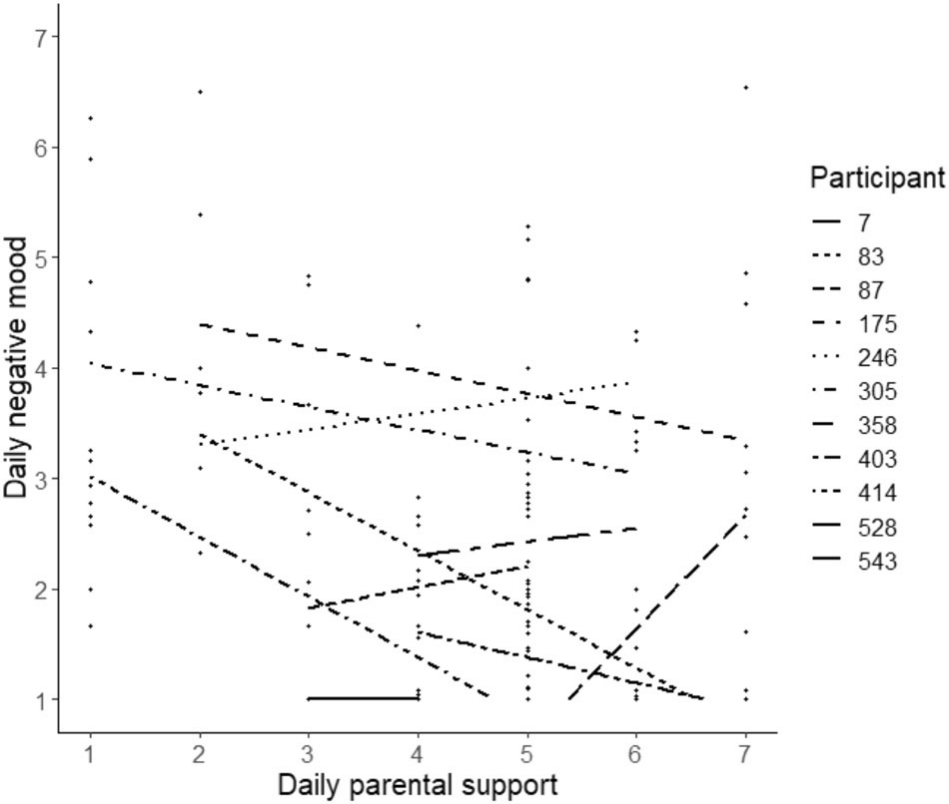
Individual-level associations between daily parental support and daily negative mood for adolescents reporting clinical levels of depressive symptoms (CDI > 16). Each line represents one person

Simple slope analysis (based on SD) on moderating effects of depressive symptoms, shown in Fig. 4, indicated that daily parental support was significantly related to daily negative mood for adolescents who reported depressive symptoms one standard deviation above the mean (*B* = −0.067, *p* < 0.001) or at the mean (*B* = −0.024, *p* = 0.009), but not for adolescents who reported depressive symptoms one standard deviation below the mean (*B* = 0.019, *p* = 0.185). However, when using the CDI-I cut-off scores to divide the sample into three groups: adolescents reporting little no none depressive symptoms, adolescents reporting subclinical depressive symptoms, and adolescents reporting clinically depressive symptoms (Kovacs 1992; Timbremont et al. 2008), results showed that even within these more homogeneous groups, there is still variation between adolescents in the linkages of daily parental support and daily negative mood. This is illustrated in Fig. 5 in which individual associations between daily parental support and daily negative mood were plotted for the 11 adolescents in the sample reporting clinically depressive symptoms.

**Fig. 6:**
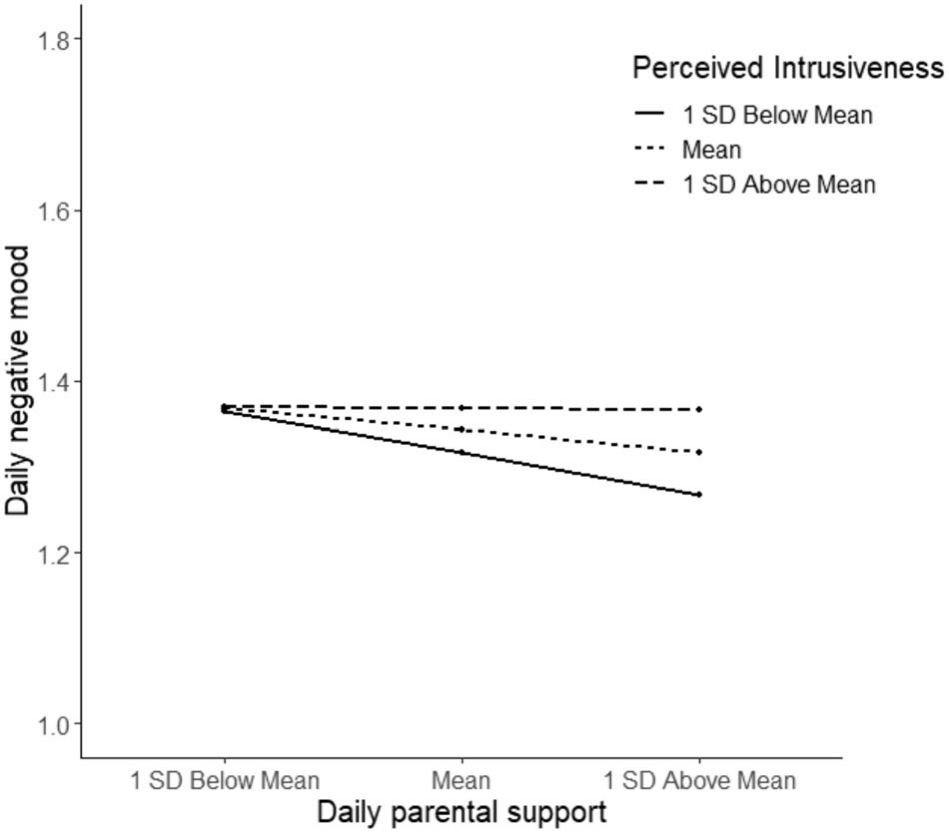
Simple slopes of daily parental support and daily negative mood for adolescents low or high in perceived intrusiveness based on (±1) Standard Deviation

Figure 6 presents simple slope analysis to interpret the significant interaction between parental support and perceived intrusiveness. Daily negative mood was significantly related to daily parental support when adolescents reported perceived intrusiveness one standard deviation below the mean (*B* = −0.045, *p* < 0.001) or at the mean (*B* = −0.023, *p* = 0.014). For adolescents who score their parents’ intrusiveness one standard deviation above the mean (*B* = −0.001, *p* = 0.970), no link was found between daily parental support and adolescent daily negative mood. Here as well, there was still variation between adolescents within the groups.

## Discussion

During adolescence, parents and parental support remain to be of key importance for adolescents’ emotional well-being (e.g., Furman and Buhrmester 1992) and many studies have shown that a lack of parental support is related to internalizing problems (e.g., Pinquart 2017). However, to date, most research used classical retrospective self-report measures, longitudinal designs, and focused on relative differences between persons, while parenting and adolescents’ experiences of affective states, such as negative mood, are both dynamic and can co-fluctuate and influence each other (Heller and Casey 2016; Pardini 2008) in shorter time intervals such as days. It has been suggested that, in general, a lack of parental support is related to more negative mood in daily life (Bai et al. 2016; Reynolds et al. 2016), but individual differences have not yet been examined let alone explained. Investigating the social processes at a more day to day micro-level could provide more insight into the building blocks of longer term mental health development. The current study therefore examined the daily within-person associations between perceived parental support and adolescent negative mood. Furthermore, it was tested whether adolescents differed in the association and if four characteristics (i.e., gender, adolescent depressive symptoms, perceived parental intrusiveness, and social support) could explain these individual differences.

The results showed that, on average, adolescents reported more negative mood on days when they perceived their parents to be less supportive. This asscociation differed significantly between adolescents which could be partially explained by the degree of adolescent depressive symptoms and perceived parental intrusiveness. The negative association between daily parental support and daily negative mood was stronger for adolescents who reported more depressive symptoms and for adolescents who perceived their parents as less intrusive. These findings suggest that in daily life, adolescents’ negative mood may be reduced by the provision of parental support, especially when adolescents experience depressive symptoms and when parents manage to respect the privacy needs of their child. Importantly, this study also provided the first insights into heterogeneity within sub-groups, which indicates that a group-differential approach to testing for explanations of heterogeneity does not suffice in understanding each adolescent’s daily life.

### Adolescent Negative Mood and Parental Support

Results of previous empirical studies and reviews showed that higher levels of parental support relate to less internalizing problems (e.g., Pinquart 2017), but these are often based on the analyses of relative differences between adolescents (the between-person level) and macro time intervals. Recently, is has increasingly been questioned whether these relative differences between families can be used for obtaining insights into what is going on within a specific family and at a more micro-level such as days (Hamaker 2012; Keijsers and Van Roekel 2018). The current study therefore aimed to understand the micro-social processes as they occur within a person in daily life. The finding that day to day fluctuations in parental support were negatively associated with fluctuations in adolescent negative mood is in line with previous findings at the between-person level and with the few studies that already examined the link between parental support and emotional well-being at the within-person level (Bai et al. 2016; Reynolds et al. 2016). These results suggest homology over ecological levels and time scales, in that previous findings on the between-person longer-term link between parental support and negative mood or internalizing problems do generalize to daily life within the average person.

### Individual Differences

Theoretically, the idea that every adolescent develops differently due to the person-specific interaction of personal and contextual influences is already widely acknowledged (Bronfenbrenner and Morris 2006; Sameroff 2010). Despite a wealth on studies on between-person interactions, relatively few studies have actually tested this conceptual idea that there may be also heterogeneity in the underlying processes that link parenting to fluctuations in adolescents’ affective well-being, let alone tried to explain these differences (e.g., Boele et al. 2019). Embracing the development and usage of methods in data collection (i.e., EMA) and new data analysis techniques (i.e., multilevel regression and random-intercept cross-lagged panel models) (Van Roekel et al. 2019), this study was able to detect that the association between fluctuations in daily parental support and fluctuations in daily negative mood differed between adolescents. This confirmed the hypothesis and hints that the broader theoretical idea of differential susceptibility (e.g., Pluess and Belsky 2010) or ecological models of development (e.g., Sameroff 2010) also apply to micro-social processes in daily life (Granic et al. 2003). For some adolescents, negative mood may be the result of a lack of support, while for others daily parental support may not have an impact on their daily negative mood. Although more studies are necessary to better understand this heterogeneity, it does highlight that it is a fallacy to assume that ‘one size fits all’ (Keijsers and Van Roekel 2018), when it comes to such person-environment interactions. The use of (new) methods and techniques which allow to collect intensive longitudinal data (e.g., Molenaar 2004) may enable us to gain more insight in the daily life processes and ultimately help clinical practice to better tailor prevention to the unique needs of a family, since adolescent daily negative mood can relate to internalizing problems (Maciejewski et al. 2014). The association between daily negative mood and adolescent depressive symptoms in the current study confirms this idea of negative mood being a precursor or even indicator of depressive symptoms.

### Explaining Differences between Adolescents

Driven by a need to better understand who may benefit, in the short term, most (or least) from parental support, four theoretically plausible characteristics that may explain the observed differences between adolescents were also tested. Gender, although previously found to be related to adolescent negative affect (Zahn-Waxler 2000), did not explain the differences between adolescents in the association between daily parental support and daily negative mood in this study. The current finding partly contradicts results of a previous study in which gender did moderate the association between family support and adolescent negative affect (Weinstein et al. 2006). However, this study examined gender in relation to the between-person association between family support and negative affect while the current study examined the within-person association between parental support and adolescent negative mood. Moreover, the previous study assessed family support on three time points, once per wave (Weinstein et al. 2006), instead of daily as in the current study. The findings thus suggest that, when focusing on micro-social processes in daily life, adolescent negative mood of boys and girls is not affected differently by parental support. More research is necessary to validate this finding.

Social support of others did also not explain the heterogeneity in contrast to the expectations. The finding of this study seems to underline the idea that parents remain a key source of emotional well-being for adolescents (Furman and Buhrmester 1992), independent of other sources. Although no sources of support were specified in the social support measure used in the current study and therefore could also include parents, friends may be another source for support since friendships become more important during adolescence (e.g., De Goede et al. 2009). A previous finding indicated that the association between family support and adolescent negative affect was stronger than the association between peer support and adolescent negative affect (Weinstein et al. 2006). Furthermore, the sample in the current study had a mean age of 14 years old and it is possible that social support of others would have had more impact if older adolescents were included. Developmental theories suggest that peers start having a stronger influence on adolescents from early to mid-adolescence and for instance peer support may become more protective with regard to adolescent depressive symptoms from mid adolescence onwards (Young et al. 2005).

Both adolescent depressive symptoms and perceived parental intrusiveness, however, did explain partly why the association between daily parental support and daily negative mood differed between adolescents, as expected. For adolescents who reported more depressive symptoms, daily parental support was more strongly related to daily negative mood than for adolescents who reported less depressive symptoms. This suggests that daily parental support is more beneficial for adolescents with depressive symptoms, but it may also indicate that the lack of parental support that day leaves especially adolescents who report depressive symptoms blue. However, as this study is correlational in nature, the reverse effect may also explain these results in that adolescents with higher levels of depressive feelings are more likely to have their own negative mood color the perception of parents as being less supportive. After having established a first indication of this within-person association between perceived parental support and adolescent negative mood, future research should assess the direction of effects, since many theories argue that parenting processes include bidirectional effects between parents and children (e.g., Bronfenbrenner and Morris 2006), for instance by examining lagged within-person effects between parental support and negative mood in daily life. Additionally, conducting a similar study in a clinical sample of adolescents could further strengthen the interpretation. Despite the additional research needed, the current findings do suggest that parenting advice which is directed at the provision of parental support should be tailored to the unique characteristics of the adolescent (i.e., adolescent’s level of depressive symptoms), as well as the processes within the specific family.

Above and beyond adolescents’ depressive symptoms, parental perceived intrusiveness also explained differences between adolescents in the association between daily parental support and daily negative mood, as expected. Compared to adolescents who reported more parental intrusiveness, for adolescents with generally non-intrusive parents, daily parental support was more strongly related to daily negative mood, suggesting that these adolescents feel better at days with more parental support. For adolescents with perceptions of privacy invasive parenting, no association between daily parental support and daily negative mood was found. Parental intrusive behaviors, such as snooping or prying into a child business, may interfere with adolescents’ normative developmental needs to establish a more autonomous position from their parents, establish privacy boundaries, and become emotionally more independent (e.g., Hawk et al. 2008). The provision of support by parents might only be effective and contribute to adolescent well-being, when parents provide support in an autonomy supportive manner (e.g., Van der Giessen et al. 2014). In fact, a recent study suggested that privacy invasion may reduce the quality of the parent-child communication, and that children undertake active measures to keep an intrusive parent more distant (Dietvorst et al. 2017). Moreover, it aligns with theoretical ideas regarding overinvolved parenting showing negative, rather than positive outcomes for the child in the longer run (e.g., McLeod et al. 2007).

However, despite the fact that adolescent depressive symptoms as well as perceived intrusiveness may explain heterogeneity, this group-differential explanation was far from conclusive. Even within a group of adolescents reporting clinically depressive symptoms, there still were differences between adolescents, with some reporting more negative mood on days when their parents were perceived supportive and others reporting less negative mood. These differences emphasize the importance of acknowledging heterogeneity even more and support the recent call to start using a more person-specific, idiographic approach in research instead of the more established nomothetic approach (Molenaar 2004), or group-differential approach when it comes to the study of parenting and adolescent well-being (Keijsers et al. 2016). With a multilevel method, this study sets one step in the direction of describing the factors that contribute to uniqueness of these processes, as well as visualizing the remaining uniqueness of each person within subgroups.

For a translation into clinical practice the current approach may open up some first insights into how to tailor interventions, but it may not suffice. Ultimately, to truly understand, each individual family may need to be studied as a unit by itself, for instance to personalize interventions to the family-specific dynamics. In the clinical practice, this more person-centered approach is already more often used (e.g., Wichers et al. 2011), leading to a burst of studies and clinical novel applications in clinical practice (e.g. Van Roekel et al. 2017). However, there is a strikingly sparsity in studies on family-specific dynamics through which parenting affects adolescent well-being (Boele et al. 2019).

### Limitations

Some limitations need to be taken into account. The sample of the study was rather homogeneous in terms of background characteristics because only adolescents of one preparatory secondary school in the south of the Netherlands participated, although the percentage of depressive symptoms in the sample aligned with prevalence percentages in the Netherlands (Statistics Netherlands 2019). It is unknown whether the current findings generalize to more ethnically diverse samples and this should be addressed in future studies. Furthermore, the study focused on short term associations and was correlational in nature, so the direction of the association or long-term effects remain unclear. Moreover, the current study focused solely on adolescent reports and perceptual biases might have affected the findings. Adolescents who show more depressive symptoms might have a more negative way of looking at their environment, also known as a negativity bias (i.e., Platt et al. 2016). This can affect their way of reporting and explain stable between-person differences in perceived privacy invasion for instance. Also, viewpoints of adolescents and parents on parenting behavior can differ. A multi-method approach such as including parental reports or observations would enable us to examine this possible perceptual bias. In addition, a suggestion for future research would be to also assess possible discrepancies in reports of parents and adolescents on for instance parental support. It has been suggested that discrepancies on for instance parent-child negative interactions influence depressive symptoms in adolescents (Nelemans et al. 2016). With regard to the measures, the measure of general social support did not differentiate between sources of social support and could also involve parental support. This could imply a possible overlap with the daily parental support measure. However, the content of the measures and the use of different time scales (daily or once) and the low correlation seem to indicate minimal overlap. Furthermore, the current study used a novel daily parental support measure that used only one item to reduce burden on the participants. To assess validity, a CFA was performed and results showed significant positive correlations with the subscale support of the NRI. Future research, using more extended scales for daily assessments would provide opportunities to examine the psychometric properties more in-depth. Lastly, parental support in general was examined instead of differentiating between maternal and parental support. According to the family system theory (Cox and Paley 1997), the mother-adolescent relationship and father-adolescent relationship can be seen as separate subsystems within a family (Restifo and Bögels 2009). Fathers and mothers might affect their adolescents differently, which could be assessed in future studies, to obtain a better understanding of the unique patterns and processes in each family.

## Conclusion

Previous studies have suggested that a lack of parental support is related to more internalizing problems in adolescents and daily negative mood has been shown to be a precursor for the development of such problems. By using EMA and daily diaries, the current study aimed to elucidate the association between perceived parental support and adolescent negative mood at the within-person level in daily life and examined to which extent adolescents would differ in this association. For the average adolescent, more negative mood was reported on days when they perceived their own parents as less supportive, which was interpreted as a protective role of parental support in preventing negative mood. However, this within-person association differed between adolescents. The negative association between parental support and negative mood in daily life was stronger for adolescents who reported more depressive symptoms, and for adolescents who perceived their parents as respecting of their privacy. The current findings demonstrated that one size does not necessarily fits all and shed new light on when a certain adolescent might be at risk for a more negative mood. Ultimately, understanding the unique micro-social processes appear to be highly informative to tailor preventive interventions for families and adolescents.

## Supplementary information

Supplementary Materials
