# Supplementary material for: The Link between Parental Support and Adolescent Negative Mood in Daily Life: between-Person Heterogeneity in within-Person Processes
Source: J Youth Adolesc. 2020 Sep 30;50(2):271–85. doi: 10.1007/s10964-020-01323-w (PMC7875844; doi:10.1007/s10964-020-01323-w)
Supplement: Supplementary file 1 — Supplementary Materials [file 10964_2020_1323_MOESM1_ESM.docx]

**Supplementary Materials**

**The Link Between Parental Support and Adolescent Negative Mood in Daily Life: Between-Person Heterogeneity in Within-Person Processes**

*Journal of Youth and Adolescence*

Loes H. C. Janssen^1,2^, Bernet M. Elzinga^1,2^, Bart Verkuil^1,2^, Manon H. J. Hillegers^3^, and Loes Keijsers^4^

*^1^Department of Clinical Psychology, Leiden University, Leiden, the Netherlands; ^2^Leiden Institute for Brain and Cognition (LIBC), Leiden, the Netherlands; ^3^Department of Child and Adolescent Psychiatry/Psychology, Erasmus University Medical Centre–Sophia Children’s Hospital, Rotterdam, Netherlands; ^4^Department Developmental Psychology, TSB, Tilburg University, Tilburg, The Netherlands*

Contact corresponding author [l.h.c.janssen@fsw.leidenuniv.nl](mailto:l.h.c.janssen@fsw.leidenuniv.nl)

**Appendix 1.**

Univariate Models for Parental Support

|  |  |  | Model fit | | |  | Range of standardized factor loadings | |
| --- | --- | --- | --- | --- | --- | --- | --- | --- |
| Instrument | Wave | *n* | CFI | RMSEA | SRMR |  | Lowest | Highest |
| NRI support | 1 | 232 | 0.962 | 0.104 | 0.059 |  | .673 | .923 |
|  | 2 | 186 | 0.963 | 0.111 | 0.068 |  | .697 | .936 |
|  | 3 | 181 | 0.927 | 0.147 | 0.064 |  | .759 | .968 |
| Daily parental support | 1 | 231 | 0.962 | 0.064 | 0.045 |  | .585 | .851 |
|  | 2 | 169 | 0.939 | 0.096 | 0.072 |  | .636 | .860 |
|  | 3 | 156 | 0.973 | 0.064 | 0.048 |  | .716 | .865 |
| NRI & daily parental support | 1 | 249 | 0.940 | 0.072 | 0.083 |  | .577 | .925 |
|  | 2 | 210 | 0.925 | 0.085 | 0.105 |  | .629 | .939 |
|  | 3 | 197 | 0.917 | 0.086 | 0.079 |  | .713 | .954 |

Note. Input and output files are available upon request.

**Appendix 2**

Model Comparisons Between Tested Model 1 to 3 (Observations = 2201)

|  |  |  |  |  | ΔLL test | | | |
| --- | --- | --- | --- | --- | --- | --- | --- | --- |
| Title | Observations | LL | AIC | BIC | Comparison | Δ-2LL | *df* | *p* |
| M1 unconditional | 2201 | -1642.984 | 3293.969 | 3316.755 |  |  |  |  |
| M2 fixed predictor daily parental support | 2201 | -1636.797 | 3283.595 | 3312.078 | M2 vs M1 | 12.374 | 1 | < .001 |
| M3 random predictor daily parental support | 2201 | -1615.370 | 3244.655 | 3284.531 | M3 vs M2 | 42.940 | 2 | < .001 |

**Appendix 3**

Model Comparisons Between Tested Model 1b to 5 (Observations = 2105)

|  |  |  |  |  | ΔLL test | | | |
| --- | --- | --- | --- | --- | --- | --- | --- | --- |
| Title | Observations | LL | AIC | BIC | Comparison | Δ-2LL | *df* | *p* |
| M1b unconditional | 2105 | -1576.952 | 3161.905 | 3184.513 |  |  |  |  |
| M2b fixed predictor daily parental support | 2105 | -1571.561 | 3153.122 | 3181.382 | M2b vs M1b | 10.783 | 1 | .001 |
| M3b random predictor daily parental support | 2105 | -1552.297 | 3118.593 | 3158.158 | M3b vs M2b | 38.530 | 2 | < .001 |
| M4a gender | 2105 | -1550.992 | 3119.983 | 3170.852 | M3b vs M4a | 2.610 | 2 | .271 |
| M4b adolescent depressive symptoms | 2105 | -1510.126 | 3038.252 | 3089.121 | M3b vs M4b | 84.341 | 2 | < .001 |
| M4c perceived social support | 2105 | -1551.656 | 3121.312 | 3172.181 | M3b vs M4c | 1.281 | 2 | .527 |
| M4d perceived intrusiveness | 2105 | -1544.391 | 3106.782 | 3157.651 | M3b vs M4d | 15.811 | 2 | < .001 |
|  |  |  |  |  |  |  |  |  |
| M5 final model | 2105 | -1506.750 | 3035.501 | 3097.674 | M3b vs M5 | 91.092 | 4 | < .001 |

**Appendix 4**

Results of Model 4a, Model 4b, Model 4c, and Model 4d on the Relation Between Daily Parental Support and Daily Negative Mood.

|  | Model 4a |  | Model 4b |  | Model 4c |  | Model 4d |
| --- | --- | --- | --- | --- | --- | --- | --- |
| Fixed effects: estimate *(SE)* |  |  |  |  |  |  |  |
| Intercept | 1.340*** (.037) |  | 1.334*** (.029) |  | 1.334*** (.037) |  | 1.333*** (.036) |
| Daily parental support | -0.031* (.012) |  | -0.023* (.009) |  | -0.031* (.012) |  | -0.031* (.012) |
| Gender | 0.120 (.076) |  |  |  |  |  |  |
| Gender*daily parental support | -0.016 (.027) |  |  |  |  |  |  |
| Depressive symptoms |  |  | 0.064*** (.005) |  |  |  |  |
| Depressive symptoms*daily parental support |  |  | -0.007*** (.002) |  |  |  |  |
| Perceived social support |  |  |  |  | 0.031 (.059) |  |  |
| Perceived social support*daily parental support |  |  |  |  | -0.022 (.021) |  |  |
| Perceived intrusiveness |  |  |  |  |  |  | 0.179*** (.051) |
| Perceived intrusiveness* daily parental support |  |  |  |  |  |  | 0.023 (.017) |
| Random effects |  |  |  |  |  |  |  |
| Between person variance | 0.231 |  | 0.124 |  | 0.234 |  | 0.219 |
| Within person variance | 0.245 |  | 0.251 |  | 0.245 |  | 0.245 |
| Random effect variance | 0.008 |  | < .001 |  | 0.008 |  | 0.008 |
| ICC | .485 |  | .331 |  | .488 |  | .472 |
|  |  |  |  |  |  |  |  |
| N individuals | 222 |  | 222 |  | 222 |  | 222 |
| N observations | 2105 |  | 2105 |  | 2105 |  | 2105 |

**p* < .05. ***p* < .01. ****p* < .001.

**Appendix 5**

Results of Model 1b, Model 2b, and Model 3b on the Relation Between Daily Parental Support and Daily Negative Mood.

|  |  | Model 1b |  | Model 2b |  | Model 3b |
| --- | --- | --- | --- | --- | --- | --- |
| Fixed effects: estimate *(SE)* |  |  |  |  |  |  |
| Intercept |  | 1.333*** (.036) |  | 1.333*** (.036) |  | 1.333*** (.036) |
| Daily parental support |  |  |  | -0.030*** (.009) |  | -0.030* (.012) |
| Random effects |  |  |  |  |  |  |
| Between person variance |  | 0.233 |  | 0.233 |  | 0.234 |
| Within person variance |  | 0.253 |  | 0.252 |  | 0.245 |
| Random effect variance |  |  |  |  |  | 0.008 |
| ICC |  | .479 |  | .480 |  | .488 |
|  |  |  |  |  |  |  |
| N individuals |  | 222 |  | 222 |  | 222 |
| N observations |  | 2105 |  | 2105 |  | 2105 |

**p* < .05. ***p* < .01. ****p* < .001.
